# Planetary Health Diet-Compatible Food Substitutions in Obesity: Direct Evidence, Gut–Organ Mechanisms, and Translational Priorities

**DOI:** 10.3390/nu18213588

**Published:** 2026-10-29

**Authors:** Minru Lin, Deju Zhang, Siyao Li, Yinuo Qu, Huiling Cai

**Affiliations:** 1 The Second Clinical College of Guangzhou University of Chinese Medicine, Guangzhou 510120, China; cccccwj169@163.com; 2 Food and Nutritional Sciences, School of Biological Sciences, The University of Hong Kong, Pokfulam Road, Hong Kong, China; u3005757@connect.hku.hk; 3 Xi’an Center for Disease Control and Prevention, No. 599 Xiying Road, Yanta District, Xi’an 710054, China; 4 Institute of Pharmacy, Shandong University of Traditional Chinese Medicine, Jinan 250355, China

**Keywords:** planetary health diet, obesity, gut microbiome, gut–organ axis, metabolic health, precision nutrition, narrative review

## Abstract

**Background:** The Planetary Health Diet (PHD) is a plant-rich reference pattern rather than a validated obesity treatment. Its clinical value is difficult to interpret when direct PHD studies are combined with evidence from related dietary patterns, isolated substrates, and experimental models. **Methods:** This narrative review searched PubMed/MEDLINE, Web of Science, Scopus, and Google Scholar through September 2026. Evidence was organized as direct PHD evidence, evidence from comparable dietary patterns or components, and predominantly experimental evidence. Human interventions and prospective cohorts were prioritized for clinical conclusions, while mechanistic studies were retained only when they clarified a PHD-compatible substitution or a testable gut–organ pathway. **Results:** Direct PHD evidence is mainly observational and supports associations with lower obesity risk, central adiposity, diabetes incidence, or metabolic dysfunction-associated steatotic liver disease; randomized studies more often test an energy-restricted program or a related plant-rich pattern. Component trials support effects on liver fat, appetite, barrier biology, and microbial function, but do not establish that a complete PHD produces the same effects. Short-chain fatty acids, bile acids, tryptophan metabolites, barrier signals, endocrine pathways, and redox/mitochondrial stress responses are plausible mediators with substantial tissue and person specificity; processing and food-safety exposures remain contextual modifiers rather than established PHD mechanisms. **Conclusions:** PHD-compatible food substitutions can be used as a clinically adaptable framework when energy intake, protein and micronutrient adequacy, cultural feasibility, affordability, and tolerability are addressed. Weight, waist, glycemia, hepatic risk, and muscle function should be evaluated separately. The complete PHD–microbiome–organ causal sequence and the added value of microbiome-guided personalization remain unproven.

## 1. Introduction

Dietary treatment of obesity has to reconcile three objectives: reducing excess adiposity, protecting organ and physical function, and sustaining an affordable eating pattern. These objectives are increasingly relevant as obesity rises across countries that also face persistent undernutrition. The NCD Risk Factor Collaboration’s pooled analysis of 3663 population-based studies documented that the growing combined burden of underweight and obesity was driven mainly by increasing obesity, underscoring the need to improve access to nutritious foods across the weight spectrum [1]. The 2025 Lancet Diabetes & Endocrinology Commission sharpened the clinical distinction between excess adiposity and illness attributable to impaired tissue or organ function. Its framework makes waist measurements, metabolic assessment, and functional limitations important complements to body mass index (BMI) [2].

The Planetary Health Diet (PHD) offers a food-system perspective on these clinical priorities. The original EAT-Lancet Commission proposed a predominantly plant-rich reference pattern that combines human nutritional needs with environmental limits. It allows the adaptation of food choices and quantities rather than requiring a single universal menu [3]. The updated Commission places healthy diets within a broader account of sustainability, access, and justice, making the feasibility of dietary change part of the intervention itself [4]. These reports establish a framework for population guidance; they do not by themselves establish comparative efficacy for treating obesity.

Prospective evidence supports taking the framework seriously. In three US cohorts, Bui and colleagues associated higher PHD adherence with lower total and cause-specific mortality and lower estimated environmental impacts. The study links long-term dietary patterns with outcomes that matter to public health, although its observational design cannot assign the benefit to a particular nutrient or microbial pathway [5]. A separate comparison of eight dietary patterns in the Nurses’ Health Study and Health Professionals Follow-Up Study found that several patterns were associated with healthy aging, defined across chronic disease and physical, cognitive, and mental domains. PHD therefore belongs within a family of healthful patterns; evidence for one does not establish its superiority over the others [6].

The gut microbiome provides a plausible connection between food selection and coordinated metabolic responses. Its relevance is functional: the same food can supply substrates for fermentation, alter enteroendocrine signals, change bile-acid transformations, or affect the intestinal barrier. In 1098 deeply phenotyped participants, Asnicar and colleagues linked habitual diet and microbial features with fasting and postprandial metabolic measures. This provides a map of candidate interactions, while leaving intervention-specific causality unresolved [7]. Among 1920 Chinese adults, repeated dietary surveys over preceding years were associated with later microbial diversity and fiber-fermenting taxa, but associations were generally small. The result supports studying long-term exposure without assuming that the microbiome is the dominant explanation for every dietary benefit [8].

**Figure 1:**
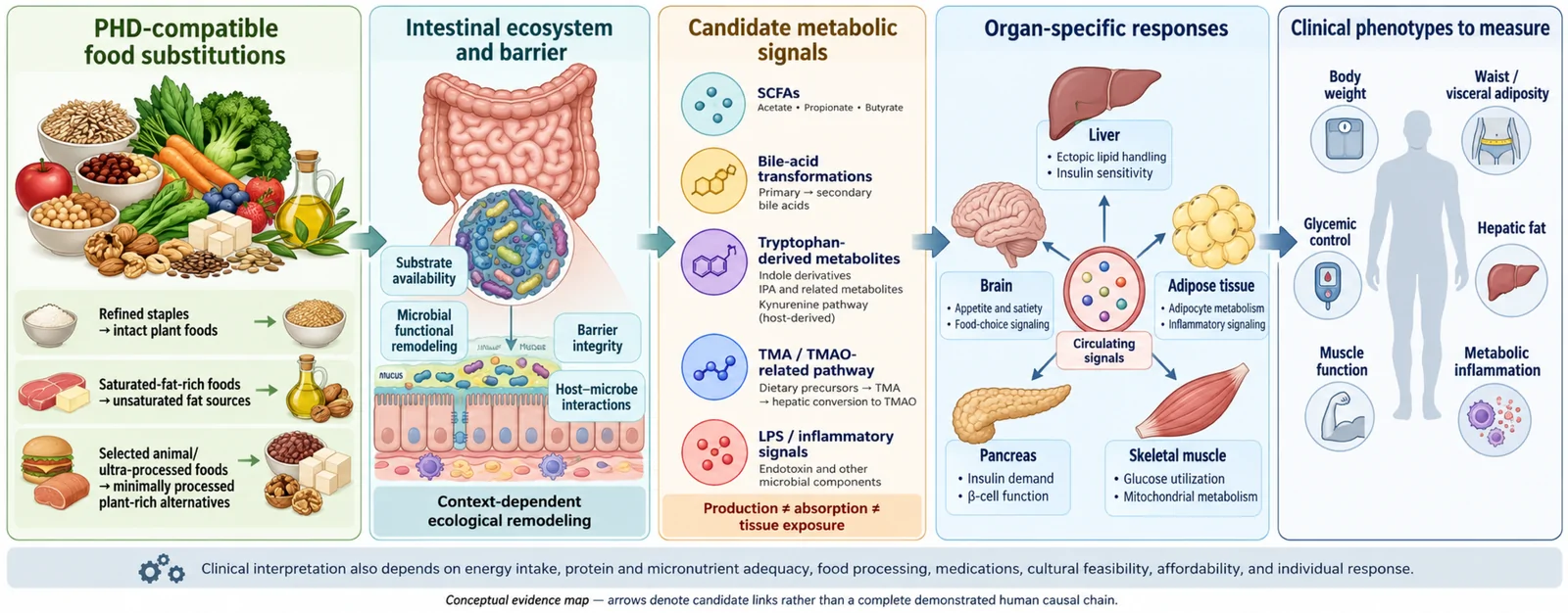
Planetary health diet-compatible food substitutions, candidate gut signals, and organ-specific outcomes. The five colored panels group food substitutions, the intestinal ecosystem and barrier, candidate metabolic signals, organ responses, and clinical phenotypes; colors do not encode evidence tiers or uniformly favorable effects. Horizontal arrows show the proposed sequence between domains, and arrows from circulating signals identify candidate organ targets. Molecular icons are schematic rather than chemical structures. Microbial remodeling is context dependent, and production, absorption, and tissue exposure are distinct. Clinical endpoints require separate measurement; arrows do not establish a complete human causal chain. The tables distinguish direct PHD evidence from related-pattern, component, and experimental evidence. Food processing is an additional exposure rather than a defining feature of every PHD index. IPA, indole-3-propionic acid; LPS, lipopolysaccharide; PHD, Planetary Health Diet; SCFA, short-chain fatty acid; TMA, trimethylamine; TMAO, trimethylamine N-oxide.

This review asks a narrower question: what can be inferred about obesity when a PHD-compatible food substitution is evaluated across clinical outcomes and candidate gut–organ pathways, while directness of evidence is kept explicit? We therefore separate direct PHD evidence from evidence on comparable dietary patterns or individual components and from predominantly experimental evidence. The clinical endpoints are visceral and hepatic fat, glycemic control, body composition, and physical function; the environmental endpoints are affordability, access, and estimated dietary impact. This structure keeps gut–organ mechanisms in service of the PHD question rather than treating them as a second, independent review. Figure 1 is an evidence map and hypothesis map, not a claim that every arrow has been demonstrated in a PHD trial.

## 2. Scope, Search Strategy, and Evidence Framework

Literature searches covered PubMed/MEDLINE, Web of Science, Scopus, and Google Scholar through September 2026. Three complementary concept groups guided the search: (i) Planetary Health Diet, EAT-Lancet diet, and PHD adherence indices in relation to obesity, adiposity, insulin resistance, and metabolic health; (ii) PHD and related Mediterranean, plant-based, and healthful dietary patterns in relation to the gut microbiome, intestinal barrier, and microbial metabolites; and (iii) obesity-related gut–organ communication, including hepatic, adipose, skeletal-muscle, neural, and pancreatic pathways. Database-specific field labels and Boolean combinations were adapted to each platform, and reference lists of relevant publications were used to identify additional studies.

Representative search strings were: (“planetary health diet” OR “EAT-Lancet” OR “planetary health diet index”) AND (obes* OR adipos* OR “insulin resistance” OR “metabolic health”); (“planetary health diet” OR “EAT-Lancet” OR Mediterranean OR plant-based) AND (microbiome OR microbiota OR SCFA OR “bile acid” OR tryptophan OR barrier); and (obes* OR “metabolic disease”) AND (“gut-liver” OR “gut-brain” OR “gut-muscle” OR “gut-adipose” OR “gut-pancreas”) AND (metabolite OR microbiome OR permeability OR redox OR mitochondrial OR ferroptosis OR mycotoxin). The searches were intentionally broad enough to identify mechanistic comparators, but an article was retained for the present review only when it informed a PHD-compatible substitution, a defined obesity-related endpoint, a food-safety constraint, or a pathway that could be tested in a PHD intervention.

Records were selected for their contribution to a defined clinical or mechanistic question, with emphasis on research published from 2021 onward and earlier studies needed to explain foundational mechanisms. We included human randomized or non-randomized interventions, prospective cohorts, cross-sectional studies with relevant obesity or metabolic endpoints, and animal, cellular, or defined-community experiments that addressed pathway specificity. We excluded studies with no PHD, related food-pattern, dietary-component, or obesity/metabolic link, and we did not treat microbiome associations without a plausible dietary exposure as evidence for PHD action. Human intervention and prospective cohort studies were prioritized for clinical interpretation; cross-sectional studies described associations; and animal or cellular experiments were used only to test biological plausibility. Reviews and consensus reports provided context rather than effect estimates. Overlapping publications from the same cohort or trial were interpreted together.

The evidence was assigned three pragmatic tiers. Tier 1 (direct PHD) included studies that measured an EAT-Lancet/PHD adherence index or assigned a PHD-oriented intervention. Tier 2 (comparable pattern or component) included Mediterranean, vegan, plant-based, fiber, resistant-starch, food-processing, metabolite, or defined-food interventions that share relevant PHD substitutions but do not test the complete PHD. Tier 3 (experimental pathway) included animal, cellular, organoid, or defined-microbial-community work used to establish pathway specificity. The tables label these tiers, and conclusions about treatment or causality are based primarily on Tier 1 human evidence. This narrative synthesis weighs exposure definition, study design, endpoint validity, consistency, and applicability; it does not estimate a pooled treatment effect or assign formal systematic-review certainty grades. Bibliographic identifiers and the source information supporting quantitative statements were checked during manuscript preparation.

## 3. What the PHD Changes: Food Substitutions, Substrates, and Dietary Context

### 3.1. An Adherence Score Is an Exposure Measure, Not a Complete Prescription

The PHD emphasizes vegetables, fruits, whole grains, legumes, nuts, and unsaturated fats, with smaller contributions from selected animal-source foods. Cacau and colleagues developed the Planetary Health Diet Index (PHDI) to translate EAT-Lancet recommendations into an assessable dietary exposure. Its food-group and energy-contribution approach is useful for population comparisons, but a similar total score can conceal different substitutions and nutrient profiles [9]. This matters mechanistically: exchanging refined cereal for intact grain changes fermentable substrate and food structure, whereas exchanging butter for plant oil primarily changes fatty-acid composition. An aggregate score cannot establish that the same gut pathway changed in both cases.

PHDI scores and other EAT-Lancet adherence scores are related but not interchangeable. Studies differ in the number and definition of food groups, whether targets are expressed as absolute amounts or energy shares, how sex-specific cut points are constructed, and how dairy, fish, tubers, added fats, red meat, and processed foods are handled. Country-specific adaptations can also change the attainable range and the meaning of a one-point difference. Accordingly, score estimates are compared within their original study rather than pooled as if they measured an identical exposure; the term “higher adherence” is preferred when the scoring system is not identical.

The quality of plant foods also matters. In 705 participants from the Baltimore Longitudinal Study of Aging, a healthful plant-based index was associated with greater microbial evenness, several polysaccharide-degrading species, and lower circulating trimethylamine N-oxide (TMAO). An unhealthful plant-based index showed opposing or null associations. Thus, the label “plant-based” alone is an inadequate description of the biological exposure [10]. In a South Asian population, a healthful plant-based pattern was likewise associated with more favorable cardiometabolic risk factors, supporting attention to culturally relevant food quality rather than the simple exclusion of animal foods [11].

Large-scale microbial comparisons reinforce this distinction. Fackelmann and colleagues analyzed 21,561 individuals across five cohorts and found recognizable vegan, vegetarian, and omnivorous microbial signatures. Features associated with favorable cardiometabolic markers were also present in omnivores who ate more plant foods. These data support a graded food-substitution approach, while their observational design does not justify prescribing a specific microbial signature as a treatment target [12].

### 3.2. Fiber Diversity Matters More than an Assumed Universal Increase in Diversity

Whole grains, legumes, vegetables, and fruits deliver carbohydrates that differ in accessibility, viscosity, and microbial utilization. Their effects depend on habitual intake and the enzymes carried by the recipient community. In Wastyk and colleagues’ randomized study, a high-fiber diet increased microbial carbohydrate-degrading capacity without increasing community diversity or improving the prespecified cytokine response score. Fermented-food intake produced a different pattern, with increased diversity and lower inflammatory markers. The study directly challenges the assumption that every beneficial dietary change should increase the same ecological measure [13].

Lancaster and colleagues showed that purified fibers also produce distinct responses. Arabinoxylan was associated with lower LDL cholesterol and altered bile acids; long-chain inulin increased *Bifidobacterium*, but its highest dose was accompanied by inflammation and increased alanine aminotransferase in some participants. This small, intensively characterized intervention supports attention to substrate identity, dose, and tolerance rather than a generic instruction to maximize fermentable fiber [14]. It also cautions against treating a food-based pattern as equivalent to high-dose isolated supplementation.

A complementary test comes from Li and colleagues’ randomized feeding study of a non-industrialized-type “restore” diet. Cardiometabolic improvements and favorable changes in several microbial metabolites occurred despite reduced microbial diversity, and benefits did not depend on administration of the accompanying *Limosilactobacillus reuteri* strain. The informative endpoint was the change in host–microbe function, not a universal direction of diversity change [15]. Together, these studies support increasing the variety of tolerated, minimally refined plant substrates while evaluating symptoms and the metabolic response.

### 3.3. Protein Source, Protein Quantity, and the Foods Being Replaced

Plant-rich diets change protein sources together with fiber, fat, energy density, and food structure. The identical-twin trial by Landry and colleagues provides useful human evidence: after eight weeks, the healthy vegan arm had lower LDL cholesterol, fasting insulin, and body weight than the healthy omnivorous arm. The randomized comparison controls some inherited and early environmental variation, but it tests an entire dietary package rather than the independent effect of plant protein or microbial mediation [16].

At the experimental level, Solon-Biet and colleagues compared protein quantity and amino-acid composition across dietary patterns and mouse diets. Their results attributed metabolic differences more strongly to total protein quantity than to the plant-versus-animal amino-acid ratios tested. They therefore weaken a simple claim that a universally favorable amino-acid profile explains plant-rich dietary effects [17]. This experiment is not a basis for lowering protein in an older adult at risk of losing muscle; nutritional adequacy and the patient’s treatment goals remain the governing constraints.

Food replacements can also change circulating metabolites without proving their causal importance. In SWAP-MEAT, 36 participants completed plant-based meat-alternative and animal-meat phases. TMAO, LDL cholesterol, and body weight were lower during the plant phase, but the TMAO response depended on intervention order, and exploratory microbiome analyses did not identify a clear responder signature [18]. The practical lesson is to evaluate the replacement product’s complete composition. These findings neither make TMAO a validated therapeutic target nor establish that every processed plant-based alternative is nutritionally equivalent to legumes or soy foods.

### 3.4. Fat Quality and Polyphenol-Rich Foods Have Both Gut-Dependent and Direct Effects

Replacing saturated-fat-rich foods with unsaturated fats can affect circulating lipids through host pathways even when microbial mediation has not been demonstrated. Eichelmann and colleagues derived a lipidomic signature from a randomized dietary-fat intervention and examined its relationship to cardiometabolic risk in additional cohorts and trial data. The work links fat substitution to measurable lipid remodeling and illustrates how an intermediate biomarker can be evaluated across study settings; it does not establish that the microbiome caused the lipidomic response [19].

Polyphenol-rich foods present a different interpretive problem. Walnuts, tea, fruit, and vegetables contain compounds that can be transformed by microbes, yet they also change fiber intake and other nutrients. The green-Mediterranean trials discussed below are valuable because they measured liver and visceral fat, but their food combinations do not isolate a single polyphenol. In a longitudinal analysis of 307 men, Wang and colleagues found that the association between Mediterranean adherence and cardiometabolic risk differed with microbial composition, including *Prevotella copri* abundance. Such interaction identifies a candidate response modifier; it does not yet provide a validated test for selecting a diet [20].

### 3.5. Food Processing Should Be Recorded Alongside PHD Adherence

A diet can meet selected plant-food targets while retaining highly processed staples, snacks, or substitutes. Hall and colleagues’ inpatient crossover experiment demonstrated that an ultra-processed diet increased ad libitum energy intake by approximately 508 kcal/day relative to an unprocessed diet, with corresponding weight gain over two weeks. The trial establishes a dietary effect on intake and weight under controlled conditions, but it does not identify a microbiome-mediated mechanism [21]. The later UPDATE crossover trial extended this comparison to diets designed to follow UK healthy-eating guidance. Both patterns produced weight loss, with greater loss on the minimally processed diet, showing that food processing can remain relevant within an otherwise health-oriented prescription [22].

Selected additives warrant a narrower inference. In a controlled feeding study of 16 adults, carboxymethylcellulose altered the fecal microbiome and metabolome, with marked mucus-layer encroachment in two exposed participants. Its short duration and specific additive dose preclude extrapolation to every processed food or to long-term obesity outcomes [23]. Similarly, Suez and colleagues’ randomized trial in 120 adults found sweetener-specific and person-specific microbiome and glycemic responses; transferring donor microbiomes into mice provided experimental support for microbial involvement. These findings identify heterogeneity, rather than a single response to all non-nutritive sweeteners [24].

Chen and colleagues’ processing-centered review positions metabolic dysfunction-associated steatotic liver disease (MASLD) as a possible amplifier of gut-derived signals relevant to brain aging. Its value here is conceptual: recording processing-related exposure alongside PHD adherence may help distinguish nutrient substitution, food-matrix effects, and hepatic consequences. It does not demonstrate that PHD prevents cognitive decline through the liver [25]. Table 1 maps these dietary choices to the evidence and endpoints needed to interpret them.

## 4. The Intestinal Ecosystem: From Food Substrates to Metabolic Signals

### 4.1. Microbial Function Is More Informative than a Universal Obesity Signature

The mechanistic question is which microbial activities respond to a PHD-compatible food pattern and subsequently alter host physiology. A single taxonomic definition of an “obese microbiome” is insufficient. In 1425 participants spanning population and gastrointestinal disease cohorts, Bolte and colleagues associated plant foods and fish with short-chain fatty acid (SCFA)-producing commensals and nutrient-metabolism pathways, whereas processed-food patterns correlated with endotoxin-synthesis potential. These associations identify plausible functions for dietary intervention; they do not establish that the same taxa mediate weight loss in every population [28].

Strain resolution changes this interpretation. A cross-cohort analysis of 8117 metagenomes identified diabetes-associated species and within-species variation that helped explain differences in metabolic risk [29]. Genome-level co-abundance analysis further identified competing microbial guilds distinguished by fiber fermentation and butyrate production versus virulence and antimicrobial-resistance features [30]. Such findings support measuring microbial functions and community interactions alongside individual organisms. Neither a diversity index nor the abundance of a single genus is an adequate surrogate for clinical benefit.

This response variability has a temporal dimension. Experiments combining human supplementation with microbial cultures showed that previous carbohydrate exposure altered subsequent inulin utilization through rapid transcriptional and community changes [31]. Alongside the substrate-specific responses discussed in Section 3, a stable baseline diet and repeated sampling are consequently more informative than comparing two isolated stool samples. Gradual implementation may be practical, but microbial adaptation itself does not establish the optimal clinical titration schedule.

### 4.2. Barrier Integrity Links Luminal Ecology to Host Inflammation

Barrier function offers a plausible connection between fermentable substrates and systemic metabolism. In mice colonized with a defined human microbial community, fiber deprivation shifted microbial resource use toward host mucus and increased susceptibility to an enteric pathogen [32]. This experiment establishes a substrate-dependent mechanism; it does not imply that all adults with obesity have pathological intestinal permeability. The original metabolic-endotoxemia experiments showed that sustained low-dose lipopolysaccharide exposure increased adipose inflammation, liver triglycerides and hepatic insulin resistance in mice [33]. They provide a causal experimental model for inflammatory amplification, while human dietary studies require direct barrier or inflammatory measurements.

### 4.3. Metabolite Production, Absorption and Tissue Exposure Must Be Distinguished

Fecal metabolite concentrations represent the balance of production, microbial consumption, host absorption and intestinal transit. They cannot be read directly as systemic exposure. In a stable-isotope study of 12 healthy adults, approximately 36%, 9% and 2% of colonic acetate, propionate and butyrate, respectively, reached the systemic circulation [34]. These different recovery fractions help explain why higher stool SCFAs need not indicate stronger signaling in muscle or brain. Claims about a metabolite-mediated dietary effect are strengthened by paired stool and blood measurements, preferably with temporal evidence linking exposure to physiological change.

The same substrate can generate chemically and biologically distinct products. Defined-community experiments showed that fiber-derived monosaccharides suppressed *Escherichia coli* indole production, leaving tryptophan available for *Clostridium sporogenes* to generate indolelactic and indolepropionic acids [35]. This mechanism depends on cross-feeding and metabolic regulation, not simply on counting tryptophan-metabolizing organisms. Bile-acid signaling is equally context dependent: an inulin diet induced microbiota-dependent cholic-acid production and type 2 inflammation in mouse barrier tissues [36]. Thus, an increase in fermentation or a shift in bile acids should be interpreted by the specific products and host response.

Finally, circulating metabolite profiles integrate several sources. In 1368 extensively characterized individuals, the analysis of 1183 plasma metabolites attributed different components of variation predominantly to diet, microbiota or genetics [37]. A metabolomic association with PHD adherence is therefore a starting point for mechanistic testing. Demonstrating microbial mediation additionally requires evidence that diet changes the relevant microbial pathway, that tissue exposure changes, and that this change contributes to an outcome.

**Figure 2:**
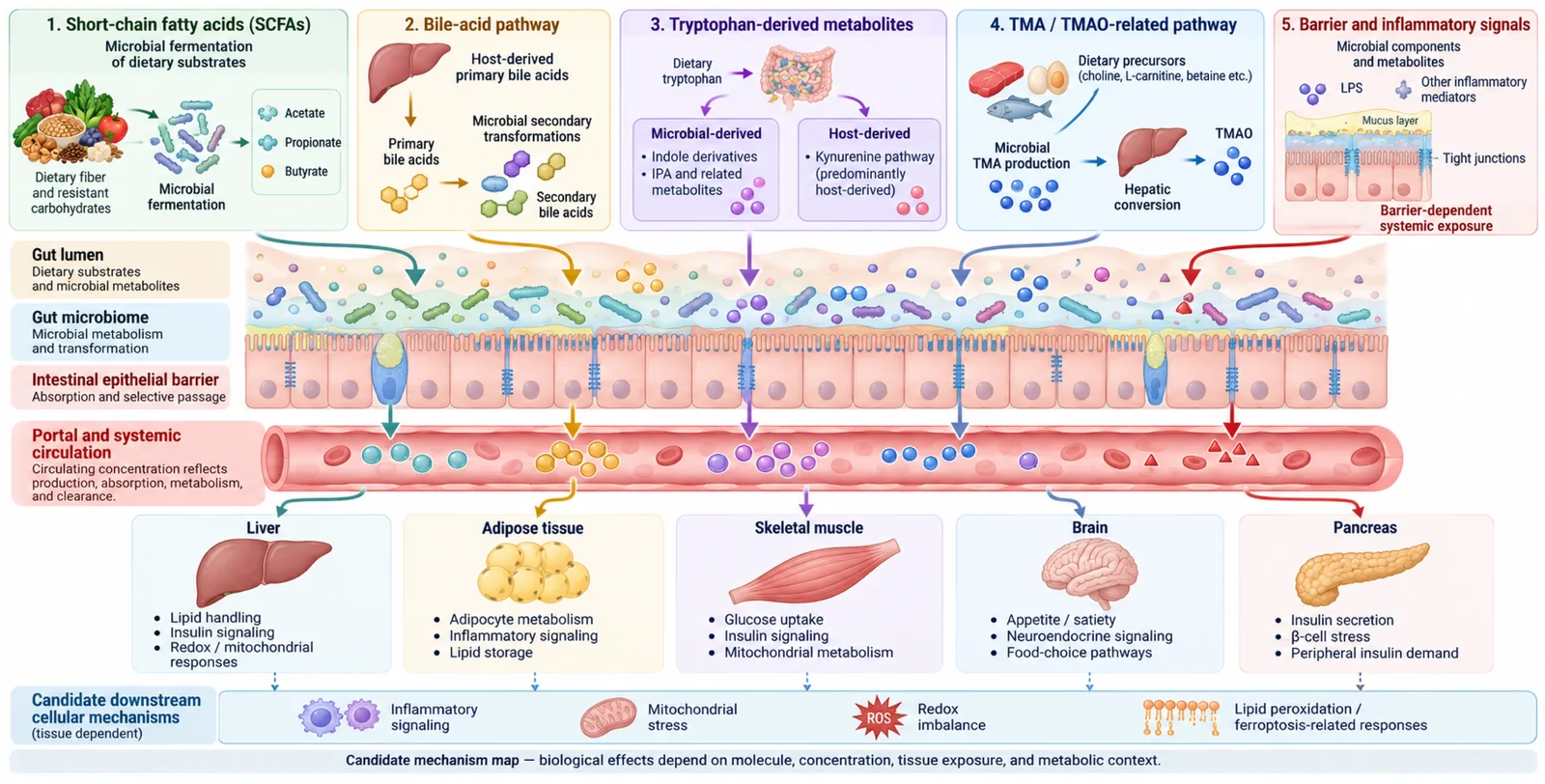
Candidate microbial and intestinal signals in gut–organ communication. Teal, orange, purple, blue, and red arrows distinguish SCFAs, bile-acid transformations, tryptophan-derived metabolites, the TMA/TMAO pathway, and barrier/inflammatory signals, respectively. Solid arrows schematically connect signal generation, epithelial passage, circulation, and candidate organ responses. Their placement next to particular organs is illustrative and does not imply exclusive one-to-one pathway specificity. Dashed arrows connect organs to tissue-dependent downstream cellular mechanisms. Colored backgrounds organize signal classes and anatomical domains rather than evidence strength or invariant benefit or harm. Primary bile acids are host-derived, secondary transformations are microbial, and kynurenine metabolism is predominantly host-derived. Circulating concentrations reflect production, absorption, metabolism, and clearance; molecular icons are not chemical structures. Biological effects depend on the molecule, concentration, tissue exposure, and metabolic context. This Tier 2/Tier 3 mechanism map does not establish mediation of a complete PHD intervention. IPA, indole-3-propionic acid; LPS, lipopolysaccharide; PHD, Planetary Health Diet; ROS, reactive oxygen species; SCFA, short-chain fatty acid; TMA, trimethylamine; TMAO, trimethylamine N-oxide.

Figure 2 summarizes how intestinal signals can reach different organ targets.

## 5. Shared Signals and Organ-Specific Translation in Obesity

The following sections organize the evidence by organ-specific clinical question after the shared issues of substrate use, barrier integrity, and systemic exposure have been addressed. Several pathways affect more than one organ, and identifying an association in blood does not identify its tissue of action. For example, reduced liver fat may coexist with persistent muscle insulin resistance, while improved glycemia may reflect reduced insulin demand rather than improved insulin secretion. Body weight, organ fat, hormone responses, and physical function therefore remain separate endpoints. Most of the studies below are Tier 2 or Tier 3 and are used to explain plausibility rather than to establish a PHD treatment effect.

### 5.1. Gut–Liver: The Strongest Dietary Mechanistic Bridge

The liver receives absorbed intestinal products and is an important site of ectopic lipid accumulation. Resistant-starch trials provide some of the clearest human evidence linking a PHD-relevant component to this organ. In a four-month randomized trial involving 196 participants with non-alcoholic fatty liver disease, resistant starch reduced intrahepatic triglyceride content by 9.08 percentage points relative to control; the difference was 5.89 percentage points after adjustment for weight loss [38]. Reductions in *Bacteroides stercoris* and circulating branched-chain amino acids (BCAAs), together with transplantation and monocolonization experiments in mice, supported a microbial contribution. Weight adjustment suggests that weight change did not account statistically for the entire difference; it does not quantify a human causal mediation effect.

A subsequent analysis, replicated in a multicenter trial, found limited resistant-starch benefit in approximately 30% of participants. Experimental work implicated competition from *Prevotella* that impaired starch utilization, whereas a selected *Bifidobacterium pseudocatenulatum* strain restored degradation in model systems [39]. This result qualifies a simple “fiber feeds beneficial bacteria” narrative. Baseline ecological interactions can prevent access to a substrate even when the prescribed dose is adequate. The clinical implication is to assess response to the intervention rather than assume benefit from reported adherence alone.

Histidine metabolism provides a second, distinct pathway. Koh and colleagues identified microbial imidazole propionate as a metabolite that impaired insulin signaling through p38γ and mechanistic target of rapamycin complex 1 in experimental systems, with supporting observations in human liver [40]. In the MetaCardis cohort, elevated imidazole propionate accompanied prediabetes and diabetes despite similar histidine intake, pointing toward altered microbial processing rather than simple precursor excess [41]. Subsequent experiments showed inhibition of metformin-related AMP-activated protein kinase signaling, with higher metabolite concentrations among metformin-treated participants with poorer glycemic control [42]. Together, these studies make drug–diet–microbiome interactions clinically relevant, while providing no basis for restricting histidine-containing foods indiscriminately.

Other liver pathways remain less directly translated. In 137 participants, lower circulating indole accompanied obesity and greater liver fat; indole administration reduced steatosis and macrophage inflammation in mice through a pathway involving myeloid PFKFB3 [43]. This supports a context-specific hepatic action, not a uniformly favorable classification of all indole products. A 12-week factorial feeding trial in people with prediabetes found improved insulin-sensitivity indices when unsaturated fat was consumed at lunch rather than dinner, alongside microbial and bile-acid changes, but no significant between-group difference in postprandial glucose [44]. The result broadens the question from nutrient composition to timing, although mediation and durable liver outcomes remain to be established.

### 5.2. Gut–Adipose: Lipid Handling and Weight Maintenance

Adiposity can change through intestinal lipid handling as well as inflammatory signaling. In a crossover trial of 37 adults with overweight or obesity, eight weeks of resistant starch produced a mean weight reduction of 2.8 kg and improved insulin resistance. Associated microbial changes were investigated in mice, where *Bifidobacterium adolescentis*, bile-acid remodeling, barrier restoration and reduced lipid absorption contributed to the response [45]. The human effect and the experimental mechanism reinforce one another, but neither establishes that an unrestricted whole PHD will reproduce the supplement’s effect size.

Microbial products can also impair lipid disposal. Liu and colleagues identified δ-valerobetaine as a microbial metabolite associated with visceral adiposity in humans. It reduced carnitine availability and mitochondrial fatty-acid oxidation experimentally, and increased adiposity and hepatic steatosis in mice in a Western-diet-dependent manner [46]. This dependence on background diet is central to the PHD hypothesis: changing the host’s nutrient environment may alter the effect of a microbial product without eliminating its producers. Conversely, oral *Blautia wexlerae* reduced diet-induced obesity and metabolic inflammation in mice after an inverse association with obesity and diabetes was identified in Japanese adults. Its effects involved several amino-acid and carbohydrate products and community remodeling [47]. These findings argue against assigning a single metabolite or genus an invariant metabolic role.

*Akkermansia muciniphila* illustrates the transition toward clinically interpretable endpoints. An initial pilot trial found improved insulin sensitivity with pasteurized bacteria, although its body-weight difference did not reach statistical significance [48]. In a 2026 randomized trial, 90 adults underwent an initial low-energy diet and then 24 weeks of weight maintenance. Regain was lower with pasteurized *A. muciniphila* than placebo, 1.2 versus 3.2 kg [49]. This is evidence for a defined adjunct after weight loss, rather than evidence that increasing fecal *Akkermansia* through any diet achieves the same result. For PHD research, maintenance after initial loss is therefore a valuable endpoint alongside short-term weight change.

### 5.3. Gut–Muscle: Insulin Sensitivity Must Be Coupled to Physical Function

Skeletal muscle creates a clinically important tension: improving insulin sensitivity while preserving protein supply and physical capacity. The foundational BCAA study linked an obesity-associated metabolite signature to insulin resistance and showed that BCAA supplementation aggravated metabolic dysfunction in rats specifically in a high-fat dietary context [50]. A later study of 277 non-diabetic adults associated circulating BCAAs with microbial biosynthetic potential; *Prevotella copri* administration increased BCAAs and worsened glucose handling in mice [51]. These findings concern metabolic context and microbial processing. They do not justify lowering essential amino-acid intake in an older person at risk of muscle loss.

Direct experimental support for a gut–muscle axis comes from germ-free mice, which exhibited muscle atrophy, altered neuromuscular-junction gene expression and impaired oxidative metabolism. Microbial transfer improved these phenotypes, while SCFAs partly reversed the impairments [52]. Human translation requires attention to the affected tissue. In 233 adults with overweight or obesity, liver and muscle insulin-resistance phenotypes differed in microbial and metabolomic features; the liver phenotype actually had higher fecal SCFAs and greater abundance of SCFA-producing bacteria [53]. This observation directly cautions against equating higher stool SCFAs with globally improved insulin sensitivity.

Urolithin A illustrates how microbial polyphenol metabolism can be studied in human interventions. In a study of 100 healthy adults, only a subset efficiently generated urolithin A after pomegranate juice, whereas direct supplementation produced more consistent systemic exposure [54]. In a randomized trial of 66 older adults, supplementation improved selected muscle-endurance measures and plasma biomarkers, but did not significantly improve the primary six-minute walking or ATP-production outcomes [55]. A separate trial in middle-aged adults found gains in muscle strength and mitochondrial-related measures without a significant improvement in the primary peak-power endpoint [56]. These trials suggest biological activity while leaving the magnitude of functional benefit unsettled; supplement exposure cannot be equated with eating ellagitannin-rich foods.

The PROMOTe trial offers an especially useful boundary. Among 72 older twins prescribed resistance exercise and BCAA supplementation, adding a prebiotic changed the microbiome and improved a cognitive measure, but did not improve the primary chair-rise outcome [57]. A microbiome response therefore cannot substitute for demonstrating preserved strength, mobility or lean tissue. PHD interventions in older adults with obesity should judge success through these outcomes as well as weight and glycemia.

### 5.4. Gut–Brain: Appetite and Food Choice, with Separate Evidence for Cognition and Activity

For the obesity question, the most direct gut–brain endpoints are appetite, satiety, food choice, and subsequent energy intake. Cognition and exercise-related behavior are relevant to long-term function but are adjacent outcomes and are discussed separately below. Gut–brain communication includes endocrine and neural pathways operating on different timescales. Targeted colonic propionate delivery stimulated glucagon-like peptide-1 (GLP-1) and peptide YY release in human colonic cells, reduced acute energy intake and attenuated weight gain in a 24-week trial of 60 adults with overweight [58]. This provides proof of principle for metabolite-linked appetite regulation using a defined delivery system. It does not imply that increasing any fermentable fiber produces an equivalent hormone response.

Food structure can affect the same hormones before colonic fermentation occurs. In a crossover pilot study of ten healthy adults, intact and disrupted chickpea meals matched for nutrients produced different upper-intestinal metabolite and hormone profiles. Intact cells prolonged GLP-1 and peptide YY release, while disrupted cells generated a sharper early glycemic response [59]. This direct nutrient-sensing pathway belongs in the PHD model because intact legumes and minimally disrupted plant tissues may influence satiety without requiring microbial mediation.

Host endocrine signaling can also reshape microbial function. Mice lacking colonic enteroendocrine cells developed hyperphagia and obesity accompanied by microbial changes that transferred the phenotype to germ-free recipients. Altered microbial glutamate production and increased food intake following colonic glutamate administration identified a reciprocal host–microbiota circuit [60]. The relevant architecture is therefore a feedback loop: intestinal cells influence the microbial signals that subsequently alter eating behavior.

Neural studies explain why subjective sweetness or taste alone does not determine preference. Duodenal neuropod cells distinguished sugars from sweeteners through different neurotransmission pathways, and sugar preference depended on glutamatergic signaling in mice [61]. Separate experiments identified gut-to-brain circuits necessary for post-ingestive fat preference, including pathways with broader nutrient sensitivity and a fat-specific component [62]. These experiments support biologically embedded food preferences, but have not shown that PHD adoption resets these circuits in humans.

Behavioral feedback may extend to activity. Dohnalová and colleagues demonstrated a mouse pathway connecting microbial endocannabinoid-related metabolites, sensory neurons and exercise-associated striatal dopamine signaling [63]. The result offers a mechanistic explanation for an interaction between gut ecology and exercise reward. Human studies must still establish whether this pathway influences adherence or activity sufficiently to affect weight maintenance.

Cognition requires a separate evidential distinction. Maternal-obesity associations with cognition in children were complemented by mouse experiments in which fiber or SCFAs improved offspring behavioral and microglial abnormalities [64]. More recently, a trial of 96 adults with obesity compared Mediterranean, ketogenic and alternate-day-fasting interventions; cognitive improvement was most evident with alternate-day fasting, accompanied by microbiota associations and supportive transplantation and microglial experiments [65]. These studies connect metabolic state with neural function across different settings. They support examining cognition in obesity research, while leaving direct PHD effects and long-term clinical significance unresolved.

### 5.5. Gut–Pancreas: Peripheral Insulin Demand and Islet Stress

Pancreatic outcomes reflect both peripheral insulin demand and intrinsic islet function. Takeuchi and colleagues combined human multi-omics with bacterial intervention in mice, linking insulin resistance to microbial carbohydrate metabolism and higher fecal host-accessible monosaccharides [66]. Selected insulin-sensitivity-associated bacteria improved mouse phenotypes. This helps specify a microbial process that could lower the demand placed on pancreatic β cells, but does not demonstrate preservation of human islet mass.

A more direct islet mechanism involves trimethylamine N-oxide (TMAO). Experimental exposure impaired glucose-stimulated insulin secretion in human and mouse islets, disrupted calcium handling and promoted stress responses; reducing TMAO production improved glucose regulation in diabetic mice [67]. Dietary interpretation requires accounting for host handling. In a randomized crossover feeding study of 113 adults, red meat increased TMAO through changes in precursor supply, microbial metabolism and renal excretion [68]. Thus, circulating TMAO is neither a specific measure of red-meat intake nor a validated standalone target for prescribing a PHD.

Other experiments show that replacing one food component may change several host pathways simultaneously. In a randomized trial of 120 healthy adults, two weeks of selected non-nutritive sweeteners produced individual microbial and glycemic responses, with transplantation experiments supporting microbial involvement [24]. This short exposure cannot resolve long-term weight-management effects. In mice, dietary sugar displaced commensals that sustained protective intestinal Th17 cells, increasing lipid absorption and susceptibility to metabolic syndrome [69]. These studies identify specific mechanisms; their clinical application depends on the replacement food, the baseline diet and the outcome being measured.

### 5.6. A Connected Model for Clinical Translation

Redox regulation offers a potential point of convergence between these organ pathways. Chen and colleagues’ synthesis links microbial signals with mitochondrial dysfunction, lipid peroxidation, and ferroptosis-sensitive injury in cardiometabolic disease [70]. A tissue-specific example comes from ergothioneine-associated transcription in proximal tubules, which opposed conserved mitochondrial injury programs and therefore suggests a candidate renal stress signature; it does not show that dietary ergothioneine or a PHD-compatible pattern is renoprotective [71]. For PHD research, this framework supports measuring a specified redox pathway alongside organ function; evidence that a whole PHD prevents ferroptotic injury in humans is still needed.

The axes converge on a common clinical sequence: intestinal exposure influences substrate handling and inflammatory or endocrine signals; liver and muscle determine ectopic lipid storage and insulin demand; adipose tissue and pancreatic function influence metabolic burden; appetite, cognition and physical activity feed back into subsequent dietary exposure. The evidence is strongest when a human intervention changes a clinically relevant endpoint and experimental work tests the associated pathway. For the PHD, this connected model remains an organizing explanation that must be tested against energy intake, food structure, protein adequacy, medication use and baseline metabolic phenotype. Its practical value is to guide outcome selection and mechanistic sampling, without making a microbiome shift the definition of treatment success.

Table 2 compares selected studies across these organ-specific pathways.

**Figure 3:**
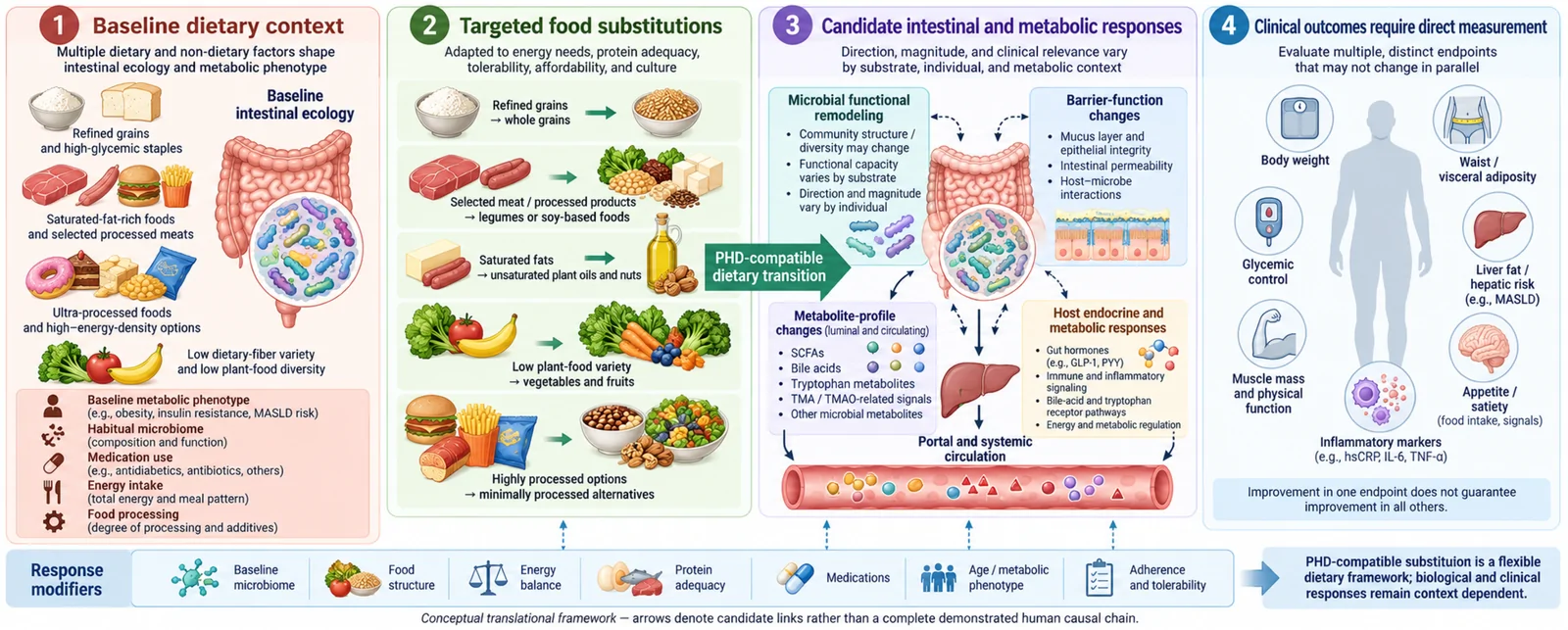
Dietary transition and candidate remodeling of the gut–organ network. The four numbered stages show baseline dietary context, targeted food substitutions, candidate intestinal and metabolic responses, and clinical outcomes requiring direct measurement. Red, green, purple, and blue backgrounds distinguish these stages; they do not represent evidence tiers or a before-and-after clinical dataset. Green arrows indicate food substitutions and the proposed dietary transition. Within the biological-response stage, solid arrows indicate candidate routes of passage or signaling, and dashed links indicate potential interactions. Dashed arrows from the lower strip identify response modifiers. Arrow direction denotes a proposed connection rather than a measured increase or decrease; molecular and organ icons are schematic. The direction and magnitude of microbial, barrier, and metabolite changes depend on the substrate, individual, and metabolic context. Improvement in one clinical endpoint does not guarantee improvement in another. GLP-1, glucagon-like peptide-1; hsCRP, high-sensitivity C-reactive protein; IL-6, interleukin-6; MASLD, metabolic dysfunction-associated steatotic liver disease; PHD, Planetary Health Diet; PYY, peptide YY; SCFA, short-chain fatty acid; TMA, trimethylamine; TMAO, trimethylamine N-oxide; TNF-α, tumor necrosis factor-α.

Figure 3 integrates these pathways into a testable dietary-transition model, linking changes in intestinal exposure to organ outcomes.

## 6. Human Evidence: From Dietary Adherence to Clinically Relevant Outcomes

### 6.1. Adiposity: Consistent Direction, Unequal Magnitudes of Association

The most useful question for obesity care is whether the Planetary Health Diet (PHD) limits excess fat accumulation while preserving function. Direct evidence has progressed from cross-sectional associations to repeated measurements of diet and body composition, although most studies still estimate associations with adherence rather than the effect of assigning the diet. Energy restriction and weight loss must be separated from dietary composition: lower energy intake can improve glycemia, liver fat, blood pressure, and inflammation regardless of whether the food pattern is called PHD, whereas composition may influence satiety, fat distribution, nutrient adequacy, and sustainability. Statistical adjustment for weight change can help test whether an association is compatible with a composition-related effect, but it can also remove part of the pathway through which food substitutions act. The randomized comparator, achieved energy deficit, physical-activity advice, contact time, and the timing of each endpoint therefore determine what can be attributed to PHD-compatible composition. In ELSA-Brasil, higher PHD Index (PHDI) adherence among 14,515 adults was associated with lower body mass index (BMI) and waist circumference; the adjusted differences were modest, despite lower odds of categorical obesity. This distinction matters because a favorable population distribution does not specify the weight loss an individual patient can expect [72]. In 27,181 US adults, higher PHDI scores similarly accompanied lower obesity prevalence and a better cardiometabolic profile. The study also linked adherence to lower estimated dietary emissions, supporting the feasibility of pursuing both objectives, but its cross-sectional adiposity analyses cannot establish their temporal sequence [73].

Prospective studies provide stronger support for prevention. Among 51,711 NutriNet-Santé participants followed for a median 8.7 years, the highest versus lowest EAT-Lancet index quintile was associated with lower incident overweight and obesity, with hazard ratios of 0.60 and 0.54, respectively. Repeated weight assessment strengthens the inference that diet quality precedes weight trajectories; voluntary participation and correlated health behaviors nevertheless limit causal interpretation [74]. The Fenland Study adds a clinically informative distinction between total weight and fat distribution. In 6845 adults, a 10-point improvement in PHDI accompanied 0.47 kg less weight gain or greater weight loss, 0.43 kg less fat mass, and 40 g less visceral fat. Associations with waist circumference, visceral fat, and liver fat score persisted after adjustment for weight change. These are concurrent observational changes, but they support measuring central adiposity alongside BMI [75].

The findings are not uniform. A Finnish analysis of 4371 adults found no association between baseline PHD score and seven-year changes in weight, BMI, or waist circumference; adherence was low and the adapted score had a narrow distribution [76]. In 44,194 Danish participants, higher adherence was unrelated to subsequent body weight after baseline adjustment, although waist circumference was slightly lower and incident obesity less frequent [77]. Neither result invalidates the positive cohorts. Together, they show that baseline adherence, score construction, the outcome chosen, and the amount of achievable dietary change influence the signal. A statistically detectable association with obesity incidence should not be translated into an assured therapeutic weight-loss effect.

### 6.2. What Intervention Studies Actually Test

A 2026 analysis of 403 adults enrolled in two controlled interventions provides more direct relevance to treatment. Both interventions prescribed a 30% energy deficit; greater PHD adherence was associated with better weight and body-composition outcomes over six months, and each 10-point increase in adherence was associated with higher odds of losing at least 10% of initial weight (odds ratio, 1.49; 95% confidence interval, 1.03–2.16). Crucially, this comparison concerns achieved adherence within interventions, rather than random assignment to PHD versus another diet. Better adherence could capture food quality, engagement, or adherence to the energy prescription [78]. The practical inference is that PHD-compatible food selection can accompany energy restriction; an independent weight-loss advantage remains to be established.

The pediatric randomized evidence reinforces the importance of the comparator. A four-month trial assigned 120 child–guardian dyads to PHD-oriented counseling, portion reduction, and activity promotion or to Brazilian dietary and activity guidance. Both groups improved children’s BMI, without a significant between-group difference; PHD scores changed little and attrition approached 40%. Thus, adding the PHD label to an active lifestyle program did not demonstrate superiority [79]. Conversely, a trial of 107 children and adolescents with abdominal obesity found improved PHD adherence and anthropometric outcomes after an intensive, moderately energy-restricted Mediterranean intervention. Here, PHD adherence was an outcome of a broader intervention, showing compatibility between patterns rather than isolating PHD efficacy [80].

### 6.3. Dysglycemia: Adiposity Explains Part, but Not Necessarily All, of the Association

The Multiethnic Cohort offers an unusually large test across ancestry groups. Among 193,379 participants, higher EAT-Lancet adherence was associated with lower obesity incidence. The type 2 diabetes hazard ratio comparing extreme tertiles changed from 0.90 to 0.97 after BMI adjustment, and the inverse association was restricted to African American and European American participants. This attenuation supports adiposity as a possible contributor while leaving room for residual confounding and differences in how the score represents customary diets [81].

In EPIC-Norfolk, 23,722 adults contributed repeated dietary assessments and a mean 19.4 years of follow-up. The highest versus lowest PHD quintile was associated with a diabetes hazard ratio of 0.68 after adjustment that included adiposity. Estimated dietary greenhouse-gas emissions were also lower. The persistence after adiposity adjustment supports investigating pathways beyond weight, but adjustment alone does not identify microbial mediation or establish a weight-independent treatment effect [82]. In MASALA, higher baseline PHDI predicted more favorable five-year glycemic, anthropometric, and blood-pressure measures in US South Asian adults. Its smaller sample and restricted setting make it most useful as evidence that culturally adapted PHD-compatible eating merits testing in a population with substantial metabolic vulnerability [83]. Across these cohorts, differences in scores, background diets, and diabetes ascertainment argue against presenting one pooled relative benefit as a universal expectation.

### 6.4. Liver and Visceral Fat: A Strong Translational Rationale with Different Evidence Sources

Direct liver evidence includes a five-year Iranian cohort of 5058 adults without baseline metabolic dysfunction-associated steatotic liver disease (MASLD). There were 562 incident cases; the highest PHDI quartile was associated with lower odds of MASLD than the lowest (0.66; 95% confidence interval, 0.50–0.88). Steatosis was assessed using controlled attenuation parameter, offering greater clinical specificity than a diet–liver-enzyme association. However, reported mediation by fiber, insulin resistance, inflammation, and steatosis-related measures should be interpreted as exploratory statistical decomposition, rather than separate established causal routes [84].

Randomized evidence for comparable plant-rich patterns supports the biological plausibility of reducing ectopic fat. In DIRECT-PLUS, 294 adults with abdominal obesity or dyslipidemia were assigned to healthy dietary guidance, Mediterranean, or green-Mediterranean diets, all with physical activity. The green-Mediterranean intervention, enriched with walnuts, green tea, and Mankai and lower in red and processed meat, produced a larger proportional intrahepatic-fat reduction than the Mediterranean comparator (38.9% versus 19.6%); the difference persisted after weight-loss adjustment [26]. A separate report from the same trial found greater visceral-fat reduction with green-Mediterranean eating (14.1%) than Mediterranean eating (6.0%) or healthy guidance (4.2%). These publications address complementary endpoints in one randomized population and should not be counted as independent replication [27]. Their results favor attention to food composition and fat distribution, but the specialized intervention and predominance of men restrict direct extrapolation to every PHD formulation.

### 6.5. Human Microbiome Findings: Candidate Pathways, Not a Validated Clinical Test

The clearest direct PHD multiomics study followed 3742 Chinese middle-aged and older adults, including 1195 with gut metagenomic measurements. Deng and colleagues identified diet-associated serum proteins, including retinol-binding protein 4 and α-2-macroglobulin, and a candidate link involving *Rothia mucilaginosa*. Repeated measurements strengthen temporal characterization, and the convergence of diet, microbiota, and circulating proteins offers hypotheses for gut–organ signaling. Nevertheless, observational mediation does not establish that changing the organism or protein will reproduce the dietary association [85].

An exploratory study of 41 healthy volunteers included only 16 who chose to follow the PHD. Over 12 weeks, α-diversity remained relatively stable; *Bifidobacterium adolescentis* increased modestly, while several other proposed favorable shifts were not significant. Self-selection, sample size, and the absence of obesity-treatment endpoints make this a feasibility signal rather than proof of microbiome restoration [86]. Comparative trials also caution against assigning an invariant benefit to a taxon. In the DIRECT-PLUS microbiome analysis, metabolic improvement with green-Mediterranean eating accompanied increased *Prevotella* and decreased *Bifidobacterium*, alongside altered branched-chain amino-acid pathways [87]. A PREDIMED-Plus substudy likewise found improved weight and metabolic measures with energy restriction and activity, but several presumed short-chain-fatty-acid-producing genera decreased. Function, substrate supply, and ecological context therefore deserve more weight than a simple “beneficial bacteria” checklist [88].

Table 3 distinguishes direct PHD studies from comparable-pattern trials and identifies which findings support prevention, treatment, or mechanistic investigation.

## 7. Clinical Translation: Match the Dietary Pattern to the Patient’s Main Risk

### 7.1. Assess Fat Distribution, Metabolic Burden, and Physical Function

Clinical implementation can begin with familiar measures: weight trajectory, waist circumference, glycemic status, blood pressure, lipids, liver and kidney status, medications, and physical function. The purpose is to define the principal treatment target and the constraints on dietary change. An individual with central adiposity and dysglycemia may need a different emphasis from an older person with obesity, low appetite, and declining strength. This is a clinical synthesis of the evidence, rather than a validated PHD treatment algorithm.

The PREDIMED-Plus body-composition substudy illustrates why weight should not be the only endpoint. Among 1521 older adults with overweight or obesity and metabolic syndrome, an energy-reduced Mediterranean diet with activity promotion reduced total and visceral fat and increased the proportion of lean mass relative to usual-care Mediterranean advice over three years. The percentage outcome must not be read as proof that absolute muscle mass increased, and the contribution of diet cannot be separated from activity and support [89]. Direct PHD evidence on muscle is less secure: a cross-sectional NHANES analysis of 9672 adults linked higher adherence to lower odds of sarcopenia and sarcopenic obesity. This is reassuring for further study but does not establish that a PHD prescription prevents muscle loss during intentional weight reduction [91]. Accordingly, adequate protein and energy planning, resistance activity appropriate to the patient, and follow-up of strength and function belong alongside fat-loss monitoring.

Kidney outcomes also favor measuring organ-specific benefit. In a post hoc PREDIMED-Plus analysis of 1909 adults, the intensive intervention attenuated decline in several estimated glomerular filtration measures; urinary albumin-to-creatinine ratio did not differ between groups [92]. A CORDIOPREV secondary analysis found a more favorable renal response to Mediterranean than low-fat eating specifically among participants with both obesity and diabetes, whereas comparable benefit was not evident across every metabolic subgroup [93]. These findings support phenotype-sensitive evaluation. They do not justify treating a population PHD food target as a renal-diet prescription; protein, potassium, energy, and medication considerations require individual clinical assessment.

### 7.2. Use Food Substitutions That Preserve Nutrient Adequacy

The most practical prescription is a short sequence of culturally familiar substitutions: legumes or soy foods for part of red and processed meat intake, whole grains for refined staples where acceptable, unsaturated fats for saturated-fat-rich choices, and fruit, vegetables, and nuts within the energy plan. Portion size and preparation remain relevant. A score can summarize direction of change, but it cannot verify that protein, essential fatty acids, or micronutrient needs have been met.

This distinction is supported by an analysis of 6835 urban residents across eight Latin American countries. Higher PHDI adherence accompanied lower inadequacy risks for several plant-associated nutrients but higher inadequacy risks for vitamin B_12_, vitamin D, and calcium. Thus, improved aggregate diet quality can coexist with specific nutritional gaps [94]. In 19,601 Latin American adolescents, higher adherence was associated with less ultra-processed food intake, yet associations with vitamin inadequacy differed between urban and rural settings. The appropriate response is to preserve nutrient-dense customary foods and use suitable fortified alternatives when needed, rather than assume that reducing animal-source foods produces an adequate diet automatically [95].

Affordability is part of clinical effectiveness. A Chilean analysis found that an EAT-Lancet-aligned food basket cost, on average, 13.9% more than the basic basket used for poverty assessment, with substantial seasonal variation. This context-specific result cannot be generalized to every setting, but it identifies a concrete failure point in counseling that ignores food prices [96]. Environmental and clinical objectives can also diverge: reducing red and processed meat may lower estimated emissions, but replacing those foods with expensive specialty products may reduce access, and reducing animal-source foods without a planned substitute can increase risks of vitamin B_12_, vitamin D, calcium, iron, iodine, or high-quality protein inadequacy. These trade-offs are especially important in older adults, people with low appetite, and patients losing lean tissue during energy restriction. Meal planning should therefore use affordable local pulses, seasonal or otherwise accessible produce, fortified foods or suitable animal-source foods when needed, familiar staple combinations, and preparation methods the household can sustain. Trials should report food cost, availability, cultural acceptability, nutrient adequacy, and environmental estimates alongside metabolic outcomes rather than assuming that a lower-emission basket is automatically the best clinical prescription.

Food safety is a further condition of a successful plant-rich prescription. A study of *Aspergillus flavus* identified a redox–lipid transcriptional state associated with high aflatoxin biosynthetic activity [97]. This is a fungal mechanistic study, not evidence that PHD adherence increases aflatoxin exposure or obesity risk. It nevertheless supports recording storage, sourcing, processing, and mycotoxin-control practices when counseling increases reliance on grains, nuts, pulses, or other crops for which contamination risk may vary by production and storage conditions. A sustainable dietary pattern must therefore be both nutritionally adequate and safe at the point of consumption.

### 7.3. Adapt Across Life Stages Without Assuming Identical Effects

During the menopausal transition, an analysis of 38,283 Nurses’ Health Study II participants found that higher PHDI adherence was associated with lower incident obesity, while a low-insulinemic pattern had the strongest association with less annual weight gain. This endpoint-specific difference favors matching advice to the food choices and metabolic problems being addressed rather than claiming that one score is uniformly superior [90]. In children, the Generation XXI cohort linked greater adherence at age seven to lower obesity and metabolic-syndrome prevalence in early adolescence. These observations support early dietary quality but need to be interpreted alongside growth, puberty, and the null comparative pediatric trial discussed above [98].

Pregnancy requires a separate inference. In a US cohort of 1464 women, higher early-pregnancy PHDI was associated with greater fetal adiposity and some lower lean-tissue measures. The study did not establish later clinical harm, but its direction shows why adult weight-prevention findings cannot be transferred uncritically to fetal development [99]. Similarly, associations of EAT-Lancet adherence with slower biomarker-based aging and longer modeled life expectancy in UK Biobank extend the potential relevance to healthy aging, but neither modeled longevity nor statistical adiposity mediation is a substitute for functional outcomes in dietary trials [100].

### 7.4. Precision Nutrition Should Demonstrate Added Clinical Value

Genetic findings do not yet support routine genotype-guided PHD prescriptions. A UK Biobank study of 171,561 adults reported lower obesity-related morbidity with greater adherence and interactions with genetic susceptibility [101]. In contrast, CARTaGENE found favorable associations between the overall PHDI and adiposity but no mediation or moderation of polygenic obesity risk by the overall index; some component-level findings weakened after multiple-testing correction [102]. Two Finnish cohorts likewise found no convincing modification of genetic associations by PHD adherence [103]. These differences favor testing prespecified interactions in adequately powered studies rather than selecting a diet from a polygenic score today.

These genetic findings also frame the next question: whether microbiome and metabolomic information improves dietary decisions beyond clinical assessment and a careful food history (Section 8).

## 8. Translational Priorities: Causal Trials and Precision Nutrition

### 8.1. Measure the Exposure and the Response at the Appropriate Level

Precision planetary nutrition is most useful when it answers a specific decision: which feasible food substitution is likely to improve this patient’s main metabolic or functional problem? The first measurements are dietary history, adherence, symptoms, body composition where indicated, and routine clinical markers. Microbiome and metabolomic measurements should then be evaluated for added information. In the Human Phenotype Project, dietary records from 10,068 individuals predicted numerous microbial species and pathways, and many associations tracked over four years. Its simulated dietary interventions are an exploratory use of observational associations, not prospective evidence that the suggested changes improve clinical outcomes [104].

Repeated exposure assessment may be as important as a more complex assay. The Food & You cohort associated the regularity of food intake and diet quality with microbial characteristics, suggesting that a single dietary average can miss relevant temporal information. However, the ability to predict microbiome composition from food logs does not show that changing meal regularity produces the predicted clinical benefit [105]. Pope and colleagues integrated fecal metabolomics, metagenomics, and dietary data from TwinsUK and ZOE PREDICT1. Fecal metabolites captured habitual food intake and cardiometabolic risk more closely than some dietary scores. Their usefulness as exposure-sensitive measurements needs to be separated from the untested proposition that targeting an individual metabolite improves health [106].

Clinical relevance also depends on what counts as success. An exploratory analysis by Chen and colleagues associated lifestyle-related blood metabolic pathways with physical and cognitive performance in aging cohorts. This offers an adjacent example of connecting circulating measurements to function, while requiring independent longitudinal validation before clinical use [107]. For PHD interventions in older adults, improvement in a metabolic signature should accompany preservation of strength, mobility, and adequate intake. A more favorable laboratory profile with worsening physical function would be an incomplete treatment success.

### 8.2. Test the Benefit of Personalization Itself

Randomized comparisons show why predictive performance and treatment value are separate questions. In Bermingham and colleagues’ 18-week trial of 347 adults, a personalized program incorporating postprandial responses, microbiome data, and health history improved triglycerides and several secondary measures relative to general dietary advice. LDL cholesterol, the other primary outcome, did not differ significantly. Because personalization was delivered as a supported, app-based package, the contribution of microbiome information cannot be isolated from the other components [108].

The PREVENTOMICS e-commerce study provides a useful counterpoint. Mediterranean adherence increased in all groups, but omics-informed recommendations did not improve general dietary habits or health parameters over control after multiple-testing correction, despite changes in selected biomarker-derived scores. More biological information therefore does not automatically produce a more effective dietary intervention [109]. A clinically useful PHD model should be tested against a realistic comparator that already includes competent dietary counseling, similar contact time, and access to appropriate foods.

**Figure 4:**
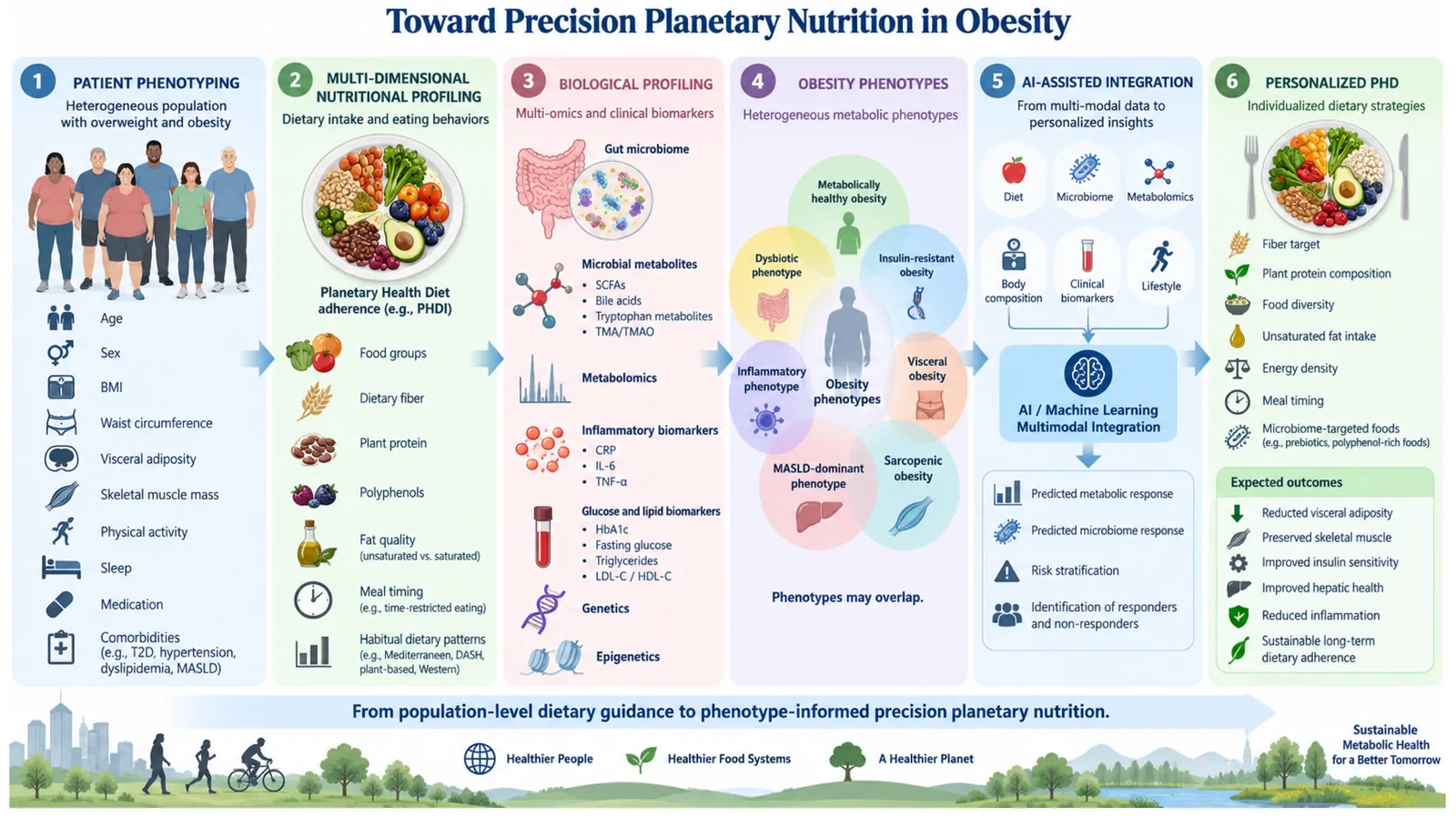
A research framework for precision planetary nutrition in obesity. Clinical phenotyping and dietary assessment can be combined with microbial, metabolomic, and other biological measurements to test response prediction. Arrows connect the proposed stages from assessment through biological profiling and model development to dietary strategies. Colored backgrounds separate measurement and decision domains, and overlapping circles indicate that the illustrated phenotypes may coexist; neither color nor overlap represents a validated diagnostic classification. Outcome icons and downward arrows identify targets to be tested rather than demonstrated treatment effects. The overlapping phenotypes are illustrative research constructs, not validated diagnostic categories. Artificial intelligence and machine learning require external validation and comparison with competent conventional dietary care; the outcomes shown are targets for testing. AI, artificial intelligence; BMI, body mass index; CRP, C-reactive protein; DASH, Dietary Approaches to Stop Hypertension; HbA1c, glycated hemoglobin; HDL-C/LDL-C, high-/low-density lipoprotein cholesterol; IL-6, interleukin-6; MASLD, metabolic dysfunction-associated steatotic liver disease; PHD/PHDI, Planetary Health Diet/Index; SCFA, short-chain fatty acid; T2D, type 2 diabetes; TMA/TMAO, trimethylamine/trimethylamine N-oxide; TNF-α, tumor necrosis factor-α.

Figure 4 presents a research framework for integrating these measurements. Development studies should keep repeated measurements from the same participant within one validation partition, evaluate performance in an external population, and distinguish prediction of adherence from prediction of treatment response. A model that predicts lower risk in people already eating well may simply recover baseline prognosis. Prespecified treatment-by-marker comparisons, calibration, and patient-relevant benefit are needed before using its recommendations in routine care.

### 8.3. Design the Next Causal PHD Trial

The most informative next trial would compare a culturally adapted PHD with an established healthy dietary pattern under comparable energy targets, behavioral support, and physical-activity advice. This would test the added value of the dietary architecture rather than a difference in counseling intensity. Repeated food assessment should describe substitutions, processing, protein adequacy, and achieved adherence alongside the score. Environmental outcomes should be estimated from the foods actually consumed and reported separately from clinical outcomes.

The mechanistic component should be organized around a prespecified chain: dietary exposure changes first, a microbial function or circulating signal changes next, and an organ-specific endpoint changes subsequently. Baseline and early follow-up metagenomics or targeted metabolites can help establish this ordering. Stool concentration alone is insufficient to establish systemic exposure; paired blood measurements, renal function, medication records, and an appropriate intestinal-barrier assessment can help distinguish production, absorption, and clearance. If a redox or mitochondrial mechanism is hypothesized, tissue-appropriate stress measures should be linked to organ function and treated as mechanistic endpoints rather than surrogate proof of benefit [70,71]. Statistical mediation should be supplemented, where feasible, by experiments on the implicated metabolite, organism, or receptor.

Phenotype selection can make such trials more informative. A MASLD-focused study needs an appropriate hepatic endpoint; a visceral-adiposity study needs a measure that distinguishes fat distribution from total weight; and a study in older adults should include muscle strength or physical performance. Chen and colleagues’ prospective multistate analysis found reciprocal associations between diabetes and sarcopenia in Chinese aging cohorts. It supports joint assessment of glucose regulation and muscle health, although its modeled transitions cannot establish that a dietary intervention prevents either condition [110]. For plant foods with relevant storage risks, the protocol should also document sourcing and food-safety controls rather than treating availability as a purely economic variable [97]. Follow-up should capture tolerability, attrition, cost, nutrient adequacy, and weight regain, because an intervention that cannot be maintained or safely delivered has limited clinical reach.

## 9. Conclusions

The PHD provides a practical framework for aligning plant-rich dietary choices with metabolic health and environmental goals, but its value in obesity should be stated at the level supported by the evidence. Direct human PHD studies are predominantly observational and support associations with lower adiposity, diabetes risk, and MASLD risk in some populations. Trials of comparable patterns and selected substrates provide evidence that defined dietary changes can affect liver fat, appetite, microbial function, and metabolic signaling, while experimental studies clarify possible pathways. These tiers support a testable gut–organ hypothesis but do not establish that the complete PHD–microbiome–organ pathway causes clinical improvement.

For current care, the useful priorities are familiar and measurable: improve food quality through feasible substitutions, plan an appropriate energy intake, preserve protein and micronutrient adequacy, assess visceral and hepatic risk, and protect muscle function. Sustainability should be pursued together with affordability, cultural fit, access, and food safety. Redox, mitochondrial, processing, and cognitive-aging studies broaden the set of pathways that future PHD trials can measure, but they remain indirect evidence for PHD efficacy. Microbiome-guided personalization remains a research opportunity whose added clinical value must be demonstrated against competent conventional dietary care. The next advance will come from trials that connect a defined food substitution to a verified biological pathway and a durable benefit that patients can experience.

## Figures and Tables

**Table 1:** PHD-compatible food substitutions and the evidence boundary. The rows summarize Tier 2 evidence from related dietary patterns or individual components; they are not direct tests of a complete PHD-mediated causal chain.

Food Substitution	Candidate Biological Change	What the Human Evidence Supports	Clinical Implication
Intact grains, pulses, and vegetables replacing refined staples	Greater substrate variety for carbohydrate fermentation; changes in bile acids and microbial enzyme capacity	Fiber interventions show substrate-dependent responses; community diversity need not increase [13,14]	Increase tolerable food sources progressively; monitor symptoms and metabolic response. Isolated high-dose fiber is a different exposure.
Legumes, soy foods, or selected plant alternatives replacing some meat	Changes in protein, saturated fat, fiber, and microbial TMA precursor supply	Vegan and meat-substitution trials improve selected lipid and weight outcomes; TMAO effects can depend on treatment sequence [16,18]	Evaluate the full replacement food and protein adequacy. Do not equate a lower TMAO concentration with an established clinical treatment effect.
Unsaturated plant oils and nuts replacing saturated-fat-rich foods	Host lipid remodeling, with possible parallel microbial effects	A dietary-fat trial linked substitution to a lipidomic signature subsequently related to cardiometabolic risk [19]	Measure lipids and fit portions to the energy plan; microbial causality is not required to justify a beneficial fat substitution.
Polyphenol-rich plant foods within a Mediterranean-like pattern	Microbial transformation of polyphenols; altered host and microbial metabolism	Green-Mediterranean trials measured reduced liver and visceral fat, but changed several foods simultaneously [26,27]	Use achievable combinations of foods; avoid attributing a whole-pattern benefit to one compound or supplement.
Minimally processed meals replacing highly processed alternatives	Changes in eating rate, energy intake, food matrix, and microbial substrate availability	Controlled trials support effects on energy intake and weight; additive studies identify narrower, exposure-specific microbial effects [21,22,23]	Record processing separately from the PHD score and assess convenience, cost, satiety, and adherence.
High-quality plant choices replacing refined or nutrient-poor plant foods	Different microbial functions despite both patterns being plant-based	Cohort analyses distinguish healthful from unhealthful plant-based diets and identify favorable signatures among some omnivores [10,12]	Counsel concrete food choices. Neither a vegan label nor a single microbial signature certifies nutritional adequacy.

**Table 2:** Mechanistic evidence used to interpret PHD-compatible substitutions. The evidence tier is shown in the first column: Tier 2 denotes a related dietary pattern or component, and Tier 3 denotes predominantly experimental pathway evidence. Study duration, comparator, and the principal endpoint are retained where reported; none establishes mediation of the complete planetary health diet.

Pathway and Study	Design, Comparator, and Duration	Principal Endpoint or Mechanistic Finding	Clinical Interpretation
Fiber–immune response *Tier 2: component* [13]	Randomized dietary intervention; 18 healthy adults per arm; 10 weeks; fermented-food comparator.	Prespecified cytokine score: no improvement with high fiber. Secondary metagenomic endpoint: increased carbohydrate-degrading capacity; diversity did not increase.	A functional microbial response need not produce a measurable anti-inflammatory effect; baseline ecology may modify response.
Resistant starch–liver *Tier 2 plus Tier 3* [38]	Four-month placebo-controlled trial; 196 participants with NAFLD; control starch, plus mouse experiments.	Primary liver-fat endpoint: intrahepatic triglyceride difference of 9.08 percentage points (5.89 after weight adjustment). Secondary BCAA and microbial changes; mouse transfer supported plausibility.	Supports testing liver fat beyond body weight. Statistical weight adjustment and mouse transfer do not quantify human mediation.
Resistant starch–response heterogeneity *Tier 2 plus Tier 3* [39]	Prespecified responder analysis, multicentre replication, and microbial experiments; intervention duration varied by trial.	Primary response contrast: approximately 30% had limited liver-fat response. Experimental endpoint: competitive inhibition of starch utilization.	Fiber selection and response monitoring may matter more than a uniform dose; microbial prediction requires prospective clinical utility testing.
Resistant starch–adiposity *Tier 2 plus Tier 3* [45]	Eight-week randomized crossover; 37 adults with overweight or obesity; control phase, plus mouse experiments.	Clinical weight and insulin-resistance outcomes: mean weight reduction of 2.8 kg. Experimental endpoints linked response to barrier, bile acids, and lipid absorption.	A component-level efficacy signal; magnitude and durability cannot be transferred directly to a whole dietary pattern.
*A. muciniphila*–maintenance *Tier 2: defined adjunct* [49]	90 adults; low-energy run-in followed by 24-week randomized maintenance; placebo comparator.	Primary maintenance outcome: weight regain 1.2 kg with pasteurized bacteria versus 3.2 kg with placebo.	Supports a defined adjunct after weight loss; does not validate stool abundance as a treatment endpoint.
Prebiotic–muscle and cognition *Tier 2: component* [57]	Twelve-week randomized trial; 72 older twins; exercise and BCAAs in both groups; placebo comparator.	Primary chair-rise outcome: no improvement. Secondary outcomes: microbial and cognitive changes.	Assess strength and mobility directly; microbial enrichment cannot establish prevention of sarcopenic obesity.
Urolithin A–muscle *Tier 2: metabolite supplement* [55,56]	Placebo-controlled supplementation trials in older and middle-aged adults; 4 months in both trials.	Primary walking/ATP or peak-power outcomes were not significantly improved; selected secondary endurance, strength, or biomarker changes occurred.	Metabolite activity is plausible, but functional efficacy and equivalence to polyphenol-rich foods remain unsettled.
TMAO–islet function *Tier 3: experimental* [67]	Human and mouse islets, cell systems, and mouse interventions; exposure and follow-up were experimental.	Mechanistic endpoint: impaired insulin secretion and calcium handling; mouse glucose regulation improved after reducing TMAO production.	A candidate islet-stress mechanism, with no established human dietary TMAO target or proof of PHD-mediated β-cell preservation.

BCAA, branched-chain amino acid; NAFLD, non-alcoholic fatty liver disease (the trial’s original diagnostic terminology); TMAO, trimethylamine N-oxide. Primary and secondary labels follow the report when identifiable; otherwise the endpoint is described as mechanistic or exploratory. Sample sizes refer to the study populations described in the original reports.

**Table 3:** Selected human studies connecting PHD adherence or comparable plant-rich dietary interventions with adiposity, metabolic health, and the gut microbiome. The first column identifies study year and evidence tier: Tier 1 denotes direct PHD exposure or a PHD-oriented intervention, and Tier 2 denotes a comparable dietary-pattern intervention. Primary clinical endpoints are distinguished from secondary or exploratory findings where reported. Multiple DIRECT-PLUS reports derive from the same trial.

Study and Evidence Tier	Design and Participants	Dietary Exposure or Comparison	Primary and Secondary Findings	Clinical Interpretation
NutriNet-Santé, 2025 *Tier 1* [74]	Prospective cohort; 51,711 adults; median 8.7 years	Direct EAT-Lancet adherence; highest versus lowest sex-specific quintile	Lower incident overweight (HR 0.60, 95% CI 0.54–0.66) and obesity (HR 0.54, 0.45–0.63); less weight gain	Supports prevention; repeated weight assessment helps temporality. Self-selection and correlated behaviors prevent a treatment-effect interpretation.
Fenland, 2026 *Tier 1* [75]	Cohort; 6845 adults with repeated diet and body-composition assessments	Direct change in PHDI; per 10-point increase	Concurrent differences: weight −0.47 kg, fat mass −0.43 kg, visceral fat −40 g. Waist, visceral-fat, and liver-score associations persisted after weight-change adjustment	Supports assessing fat distribution beyond BMI. Concurrent changes do not establish whether dietary change caused redistribution; liver fat was represented by a score.
Finnish adult cohorts, 2023 *Tier 1* [76]	Follow-up study; 4371 adults; seven years	Direct Finnish-adapted PHD score and national recommendation score	No significant associations with weight, BMI, or waist changes; mean PHD score 3.6 of 13	A relevant null result. Low adherence and limited score variation may reduce contrast, but should not be assumed to fully explain the null findings.
Danish Diet, Cancer and Health, 2023 *Tier 1* [77]	Cohort; 44,194 for weight and 43,678 for waist; five years	Direct baseline EAT-Lancet score; 11–14 versus 0–7 points	No association with subsequent weight after baseline adjustment; waist difference −0.38 cm; obesity RR 0.89 (0.82–0.98)	Weight, central adiposity, and threshold-defined obesity can yield different conclusions. Follow-up anthropometry was self-reported.
Multiethnic Cohort, 2024 *Tier 1* [81]	Prospective cohort; 193,379 adults from five ethnic groups	Direct adapted EAT-Lancet index; highest versus lowest tertile	Obesity HR 0.76 (0.73–0.79). Diabetes HR changed from 0.90 to 0.97 after BMI adjustment	Suggests an adiposity-related contribution to diabetes associations. Ethnic heterogeneity limits universal effect estimates; BMI adjustment is not proof of mediation.
EPIC-Norfolk, 2025 *Tier 1* [82]	Cohort; 23,722 adults; mean 19.4 years; 3496 incident diabetes cases	Direct PHD score with repeated dietary assessment; highest versus lowest quintile	Diabetes HR 0.68 (0.61–0.76), including adiposity adjustment; estimated emissions approximately 18% lower	Supports joint metabolic and environmental relevance. Residual confounding remains; lower emissions were estimated from dietary data.
Energy-restriction studies, 2026 *Tier 1* [78]	Adherence analysis within two interventions; 403 adults; six months	Direct PHD adherence during a prescribed 30% energy deficit	Per 10-point higher adherence, OR 1.49 (1.03–2.16) for achieving ≥10% weight loss; favorable anthropometric associations	Supports PHD-compatible eating within weight management. Participants were not randomized to achieved PHD adherence, so comparative dietary efficacy is unresolved.
Family-based pediatric trial, 2025 *Tier 1* [79]	Randomized trial; 120 child–guardian dyads; four months	PHD counseling, portions, and activity versus Brazilian dietary/activity guidance	Children’s BMI decreased in both groups without significant between-group difference; adherence changed little; attrition about 40%	No demonstrated superiority over active lifestyle guidance. Limited intervention contrast and attrition complicate interpretation.
Chinese multiomics cohort, 2025 *Tier 1* [85]	Prospective cohort; 3742 adults; 1195 with metagenomics; repeated measures	Direct EAT-Lancet adherence assessed by food-frequency questionnaire	Four core diet-associated proteins; five microbial biomarkers; candidate *R. mucilaginosa*–α-2-macroglobulin association	Provides molecular hypotheses relevant to cardiometabolic risk. Statistical mediation cannot establish a targetable causal gut–organ pathway.
PHD microbiome pilot, 2023 *Tier 1* [86]	Nonrandomized descriptive study; 41 healthy adults; 12 weeks	Self-selected PHD (n=16) versus omnivorous (n=16) or vegetarian/vegan (n=9) diets	Stable α-diversity; modest increase in *B. adolescentis*; several other shifts nonsignificant	Demonstrates feasibility of serial microbial assessment. Small self-selected groups and no obesity-treatment endpoint limit clinical inference.
Iranian MASLD cohort, 2026 *Tier 1* [84]	Cohort; 5058 adults without baseline MASLD; five years	Direct PHDI; highest versus lowest quartile	562 incident cases; MASLD OR 0.66 (0.50–0.88); steatosis assessed by controlled attenuation parameter	Direct organ-specific association. Does not show fibrosis regression or establish the proposed fiber, inflammatory, or insulin-resistance mediation pathways.
DIRECT-PLUS liver study, 2021 *Tier 2* [26]	Randomized trial; 294 adults with abdominal obesity/dyslipidemia; 18 months	Comparable pattern: green-Mediterranean versus Mediterranean or healthy guidance; all with activity	Relative intrahepatic-fat reductions: 38.9%, 19.6%, and 12.2%, respectively; green versus Mediterranean difference persisted after weight-loss adjustment	Supports reducing ectopic fat through food-pattern interventions. Specialized foods, predominantly male sample, and incomplete follow-up imaging limit transferability.
DIRECT-PLUS visceral-fat study, 2022 *Tier 2* [27]	Additional report from the same 294-participant randomized trial	Comparable pattern: polyphenol-enriched green-Mediterranean diet with less red/processed meat	Visceral-fat reductions: 14.1% green-Mediterranean, 6.0% Mediterranean, 4.2% healthy guidance	Extends evidence to fat distribution, but is not independent replication of the liver trial. Food-component associations do not isolate individual active ingredients.
PREDIMED-Plus body composition, 2023 *Tier 2* [89]	Randomized-trial substudy; 1521 older adults; three years	Comparable pattern: energy-reduced Mediterranean diet plus activity versus ad libitum Mediterranean advice	At three years: between-group differences of −0.38 percentage points total fat, −70.4 g visceral fat, and +0.34 percentage points lean mass	Supports monitoring composition during treatment. Relative lean-mass improvement is not synonymous with absolute muscle gain; the intervention was multicomponent.
Nurses’ Health Study II, 2026 *Tier 1* [90]	Prospective cohort; 38,283 women during the menopausal transition	Direct PHDI and other dietary indices; highest versus lowest quintile	PHDI showed the lowest observed obesity HR (0.46, 0.42–0.51); a low-insulinemic pattern had the strongest weight-gain association	Identifies a clinically relevant life stage. Rankings vary by endpoint and remain observational; weight was self-reported.
NHANES sarcopenia analysis, 2026 *Tier 1* [91]	Cross-sectional analysis; 9672 adults	Direct EAT-Lancet score; highest versus lowest quartile	Sarcopenia OR 0.72 (0.54–0.95); sarcopenic-obesity OR 0.58 (0.42–0.82)	Supports testing muscle-related outcomes. Cannot establish muscle preservation during energy restriction or causal inflammatory mediation.

*Abbreviations*: BMI, body mass index; CI, confidence interval; HR, hazard ratio; MASLD, metabolic dysfunction-associated steatotic liver disease; OR, odds ratio; PHD, Planetary Health Diet; PHDI, Plan- etary Health Diet Index; RR, risk ratio. Estimates are study-specific adjusted associations or between- group comparisons as indicated; they are not directly interchangeable across score definitions. Pri- mary outcomes are reported first when the publication identified them; microbiome, biomarker, or mediation results are secondary or exploratory unless stated otherwise.

## Data Availability

No new research datasets were generated or analyzed for this narrative review. The evidence discussed is available in the cited publications. Requests for information about the literature synthesis, or for assistance with related data and code, may be directed to the corresponding authors.
